# Successful mentalizing in schizophrenia is associated with specific recruitment of ventromedial prefrontal cortex and precuneus

**DOI:** 10.1192/j.eurpsy.2022.850

**Published:** 2022-09-01

**Authors:** M. Jáni, M. Gajdoš, L. Ustohal, J. Hořínková, T. Kašpárek

**Affiliations:** 1 Masaryk University - Faculty of Medicine, Department Of Psychiatry, Brno, Czech Republic; 2 Masaryk University, Central European Institute Of Technology, Brno, Czech Republic

**Keywords:** schizophrénia, social cognition, fMRI, mentalizing

## Abstract

**Introduction:**

Patients with schizophrenia have difficulties in cognitive and affective mentalizing which is manifested by excessive (‘overmentalizing’) or defective (‘undermentalizing’) attribution of mental states. As most of the tests does not differentiate between ‘overmentalizing’ and ‘undermentalizing’, it is not yet clear how are these deficits reflected in mentalizing network in schizophrenia.

**Objectives:**

Investigate how is cognitive and affective ‘overmentalizing’ and ‘undermentalizing’ reflected in mentalizing network in schizophrenia.

**Methods:**

We recruited 30 schizophrenia patients and 30 healthy controls who underwent fMRI session while they completed ‘Social situations assessment task’ consisting of 90 stories with 30 questions on affective, 30 on cognitive mental states and 30 control memory questions and 4 possible answers: no mentalizing, undermentalizing, appropriate mentalizing, overmentalizng (for control condition 1 correct and 3 incorrect).

**Results:**

On a behavioral level, we found increased no mentalizing and undermentalizing and decreased mentalizing in patients, but no difference in overmentalizing between groups. For fMRI results, patients showed lesser recruitment of dorsomedial prefrontal cortex and temporal poles (with right superior temporal sulcus) only during appropriate mentalizing in both affective and cognitive conditions. However, ventromedial prefrontal cortex and precuneus showed increased pattern of activation across all mentalizing levels in healthy controls compared to schizophrenia, but suppressed activation in appropriate cognitive mentalizing corresponding to the level of a schizophrenia group.

**Conclusions:**

Schizophrenia patients show different pattern of mentalizing compared to healthy control that can be associated with specific activity mentalizing brain network.

**Disclosure:**

No significant relationships.

